# Analysis of Plasticizer Contamination Throughout Olive Oil Production

**DOI:** 10.3390/molecules29246013

**Published:** 2024-12-20

**Authors:** Flávia Freitas, João Brinco, Maria João Cabrita, Marco Gomes da Silva

**Affiliations:** 1 LAQV/REQUIMTE, Department of Chemistry, NOVA School of Science and Technology, NOVA University Lisbon, 2829-516 Caparica, Portugal; fs.freitas@campus.fct.unl.pt; 2 MED-Mediterranean Institute for Agriculture, Environment and Development & CHANGE-Global Change and Sustainability Institute, Institute for Advanced Studies and Research, Universidade de Évora, Pólo da Mitra, Ap. 94, 7006-554 Évora, Portugal; 3 CENSE-Center for Environmental and Sustainability Research & CHANGE-Global Change and Sustainability Institute, NOVA School of Science and Technology, NOVA University Lisbon, Campus de Caparica, 2829-516 Caparica, Portugal; j.brinco@campus.fct.unl.pt; 4 MED-Mediterranean Institute for Agriculture, Environment and Development & CHANGE-Global Change and Sustainability Institute, Departamento de Fitotecnia, Escola de Ciências e Tecnologia, Universidade de Évora, Pólo da Mitra, Ap. 94, 7006-554 Évora, Portugal

**Keywords:** olive oil, plasticizers, phthalates, contamination, production line, analysis

## Abstract

This study monitored the contamination of 32 plasticizers in olive oil throughout the production and storage process. Samples were collected at different stages of production from three olive oil production lines in distinct regions of Portugal and analyzed for 23 phthalates and 9 phthalates substitutes to identify contamination sources. The developed analytical method employed liquid–liquid extraction with hexane/methanol (1:4, *v*/*v*), followed by centrifugation, extract removal, and freezing as a clean-up step. Analysis was conducted using gas chromatography tandem mass spectrometry (GC-MS/MS), with detection limits ranging from 0.001 to 0.103 mg/kg. The results revealed that plasticizer concentrations progressively increased at each stage of the production process, although unprocessed olives also contained contaminants. Di-isononyl phthalate (DINP) was the most prevalent compound, but all phthalates regulated by the European Union for food contact materials were detected, as well as some unregulated plasticizers. In a few packaged olive oils, DINP concentrations exceeded the specific migration limits established by European regulations. Samples stored in glass and plastic bottles showed no significant differences in plasticizer concentrations after six months of storage. However, higher concentrations were observed in plastic-packaged samples after 18 months of storage. Our findings indicate that the primary source of plasticizer contamination in olive oil originates from the production process itself, except for prolonged storage in plastic bottles, which should be avoided.

## 1. Introduction

Olive oil, rich in antioxidants such as phenols and tocopherols, is essential in the Mediterranean diet and widely consumed worldwide, with a consumption of approximately three million tons per year [1,2]. Its processing occurs mechanically and/or physically, ideally without altering its chemical composition.

Initially, the materials used in the production and storage of olive oil included wood, glass, metal, and clay, but industrial evolution led to the use of polymers, such as polyethylene and polypropylene, especially in packaging, due to their advantages in cost, recyclability, and durability [3,4,5,6,7].

The versatility of polymers led to the development of additives to enhance their properties, such as phthalates, which are used to make the material more flexible or rigid, depending on the need. These plasticizers are widely used in the food and engineering industries in products ranging from food packaging to pipes, tubes, and mats [8,9,10,11]. Despite their benefits for polymer durability and functionality, phthalates have low solubility in water and high solubility in lipid matrices, such as olive oil, which may result in their migration into food products [9,10].

Several studies have indicated that these additives may pose health risks, leading to extensive research into the toxicity of phthalates [12,13,14,15,16,17,18,19,20,21,22]. As a result, several countries have intervened and regulated exposure to these plasticizers.

In Europe, food safety concerns have led to the imposition of strict requirements for food contact materials (FCMs), as specified in Regulation (EC) No. 1935/2004 [23]. This regulation prohibits materials from transferring substances to food in quantities that could harm human health or adversely affect its organoleptic properties. Specific migration limits (SMLs) have been defined for five permitted phthalates (DEHP, BBP, DBP, DINP, and DIDP, see Table 1) in Annex I of Regulation (EU) No. 10/2011, based on toxicological assessments [24]. Additionally, in 2019, the European Food Safety Authority (EFSA) established tolerable daily intake for four of these phthalates, ranging from 50 µg/kg for DBP, BBP, DEHP, and DINP to 150 µg/kg for DIDP [25].

Later, in 2023, Regulation (EU) 2023/1442 updated the migration limits to strengthen consumer protection further (Table 1) [26].

Currently, there are no specific regulations defining the permissible levels of phthalates in food. Therefore, even though migration limits are monitored in packaging, it is essential to identify the sources of these plasticizers migrating into food. Detecting contamination may indicate that the food has come into contact with unsuitable materials during the production process.

For olive oil, research indicates that phthalates may be introduced both during the production process and during the treatment of the olives. This is because these compounds are commonly found in materials such as harvesting nets, pipes, tanks, and various other plastic components. [27,28]. Additionally, storage in synthetic corks and plastic containers can contribute to this contamination. Even the drinking water used for irrigation or washing production materials may contain these plasticizers [29]. Thus, controlling and studying the use of polymers and additives in the olive oil production chain is essential to ensure food safety and consumer health.

Lastly, it is important to note that due to the restriction on the use of certain phthalates and the ongoing pressure from the scientific community, alternative compounds to these plasticizers are emerging, such as terephthalates, trimellitates, adipates, and sebacates, among others [30,31,32,33,34]. Some of these are authorized for use in the manufacture of plastic materials intended to come into contact with food and are included in the Restriction Group No. 32 of Regulation (EU) 2023/1442 (Table 1) [26]. However, the migration of these substances into food and their implications for human health are still not well understood. This highlights the need for toxicological studies to examine their impact on human health, as well as further analytical exploration for potential future regulatory controls [33,35,36,37].

Therefore, there is an urgent need to develop analytical methods that allow the identification and quantification of phthalates and phthalate substitutes at low concentrations throughout the olive oil production line [28,38].

The analysis of plasticizers in olive oil presents challenges due to the low concentrations of these compounds and the interference of the lipophilic matrix, which requires methods with adequate clean-up/separation and low detection limits [39,40,41]. It is essential to avoid contamination during laboratory handling, given the omnipresence of plasticizers in plastic materials. To ensure data reliability, stringent control measures are necessary [42,43,44,45].

Due to the complexity of the matrix, samples require prior preparation before analysis, typically by gas chromatography (GC) or liquid chromatography (LC), with mass spectrometry (MS) as the detection method. Other techniques, such as UV spectrophotometry, Raman spectroscopy, and chemiluminescence, are also employed [38].

To optimize the recovery of plasticizers and minimize interferences, different extraction and pre-treatment approaches are recommended, such as liquid–liquid extraction (LLE), solid-phase microextraction (SPME), solid-phase extraction (SPE), and the QuEChERS method, among others. These methods enable more accurate quantification of plasticizers in complex food matrices [38].

The objective of this study was to investigate contamination by 23 phthalates and 7 phthalate substitutes in olive oil throughout the production process and in packaged olive oil to ascertain where plasticizer contamination was originating. To this end, several samples were taken at different stages of the production process from three olive oil production lines. Plasticizer determination was achieved using a simple, fast, and reliable analytical method that combines LLE extraction followed by freezing. This method was employed for efficient extraction and clean-up, with reduced solvent consumption, minimizing cross-contamination from laboratory materials in order to lower background contamination levels in the analytical procedure. Separation and detection were carried out by GC-MS/MS, without the need for pre-concentration steps.

## 2. Results and Discussion

### 2.1. Analytical Method Development

For sample analysis, we intended to develop a robust method that reached low detection limits whilst also minimizing the amount of oil in the final extract. Furthermore, the entire method should be performed without recourse to plastic material in an attempt to avoid contamination. Several methods have already been developed for the analysis of phthalates in olive oil [38]. Most methods employ either a liquid–liquid extraction, often with acetonitrile and followed by some type of clean-up such as dispersive solid-phase microextraction (d-SPE), or they simply dilute the olive oil with hexane and inject it (“dilute-and-shoot”). Although liquid–liquid extraction with acetonitrile has shown adequate results, the need for extensive clean-up presents an extra step and a possible source of contamination (plastic Eppendorf tubes commonly used in d-SPE are a problem, for example). Furthermore, acetonitrile has a relatively high boiling point and thus is not amiable to pre-concentration techniques such as solvent-drying or programmed-temperature volatilization, especially when analyzing low boiling point phthalates. Simple “dilute-and-shoot” with hexane is an incredibly simple technique that greatly reduces the possibility of laboratory contamination but often requires specialized GC equipment such as pre-columns and frequent inlet liner changes due to the introduction of waxes and other low-volatility components of olive oil.

The original method that was adapted used an extraction with pentane/acetone, followed by centrifugation and removal of the supernatant. After experimentation with different solvents, it was found that hexane/methanol (1:4 *v*/*v*) was a better mixture, both because pentane was too volatile for quantitative work and methanol provided a better phase separation. The hexane reduced the extraction solvent’s polarity since only methanol was found not to adequately extract most plasticizers. The original method used 3 mL of extraction solvent twice for 0.5 g of olive oil, but we found 2 mL to be ideal, as 1 mL was operationally difficult to remove after centrifugation, and 3 mL had a lower concentration factor. After injection of an extract into a GC-FID with a high-temperature DB-5MS column, it was found that many high-boiling point compounds had been extracted; thus, a simple clean-up method was devised by freezing the samples overnight and then removing the liquid phase. In most frozen samples, a significant amount of solid precipitate was found.

Matrix-induced response enhancement for olive-oil extracts was quite significant. For some compounds, calculated plasticizer concentrations of a spiked blank using a calibration in pure hexane/methanol (1:4 *v*/*v*) were over twice the actual spiked concentration. Thus, calibrations were performed by spiking the blank oil and then performing the extraction. Simple matrix-matched calibration (by extracting the blank and spiking just before injection) would have adequately corrected for matrix effects but not for recovery. Since excellent repeatability was obtained for extraction triplicates of every spiked concentration (the highest being 9.6% RSD for DMP at 343 ng/g, and commonly between 1–5% RSD), this calibration method proved successful and more accurate. Sunflower oil was used as a surrogate matrix instead of olive oil because, during initial testing, it was found that all olive oils analyzed, including an olive oil analytical standard, contained significant amounts of plasticizers. The sunflower oil in question was selected because it was the only oil among several tested that showed no appreciable contamination by the evaluated plasticizers. Moreover, it mimics the oily nature of olive oil as well as most of its constituents, although in distinct proportions. Indeed, sunflower oil is similar to olive oil in terms of lipid composition and physical characteristics. Since calibration without some form of matrix-matching would significantly compromise the method, the choice of sunflower oil was, therefore, the best alternative available to ensure accuracy in method calibration and validation.

### 2.2. Olive Oil Production and Plasticizer Contamination

Olive oil is present as small droplets within the vacuoles of mesocarp cells in olive fruits. It is also found, in smaller proportions, within the colloidal system of the cell cytoplasm and, in even smaller amounts, in the epicarp and the endosperm [46].

**Figure 1:**
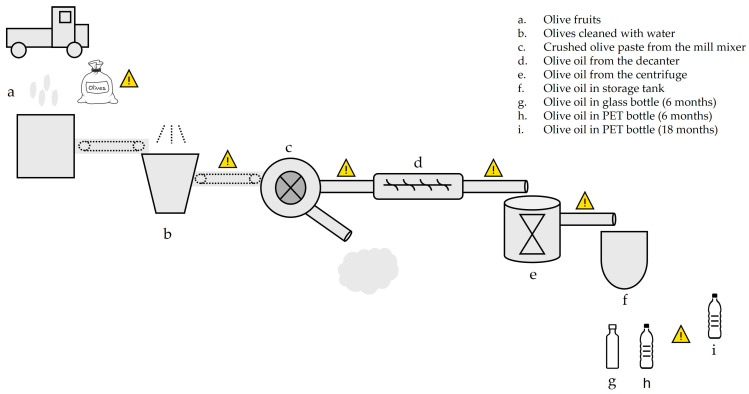
Diagram of olive oil production. The letters represent the samples taken from each step of the production process. Samples g and h were analyzed 6 months after bottling, whereas sample i was analyzed 18 months after bottling. Olive oil production follows from sample a to f. The warning symbol represents critical points of contamination during olive oil production, such as plastic bags, conveyor belts, tubes and hoses, and storage containers.

To obtain olive oil, several individual steps are required. Figure 1 shows a simplified diagram of olive oil production, as well as the different intermediate products obtained, from olives to packaged olive oil.

After harvesting, the olives are transported in baskets, containers, or plastic bags (sample a). Once they arrive at the mill, leaves, branches, and other foreign materials must be removed, followed by a thorough washing with clean water to eliminate impurities that could damage the equipment or compromise the final product’s quality (sample b). For example, the presence of leaves can impart a bitter taste to the oil.

Next, the olives are transported via conveyor belts to the next stage: crushing and malaxation (sample c). This process, carried out in the mill, aims to crush and rupture the pulp cells to release the oil stored in the vacuoles. After crushing, the resulting paste undergoes malaxation, a slow and continuous mixing process that promotes the coalescence of oil droplets, thereby increasing extraction efficiency.

After malaxation, the olive paste mainly consists of oil, small fragments of olive pits, water, and cellular residues. The next step is separating the oil from other by-products. This involves extracting the liquid phase (the mixed oil or “olive must”) from the solid phase (pomace). The oil is then transferred through hoses to a decanter, where suspended particles are removed, allowing for an initial purification (sample d). Finally, the oil undergoes a final separation in a centrifuge to remove water residues and any other unwanted substances, resulting in a product ready for consumption or storage (sample e).

After processing, the olive oil is typically stored in stainless steel tanks (sample f), which protect the product from oxidation and preserve its organoleptic characteristics. Subsequently, the oil is packaged in glass bottles (sample g), which provide excellent protection against light and external contaminants, or in polyethylene terephthalate (PET) bottles (sample h), a lighter and more economical alternative often used for oils intended for quick consumption. These containers are then prepared for commercialization, ensuring the product reaches consumers with its quality intact.

In the past, most of the utensils and equipment used in olive oil production were made from conventional materials such as stone, ceramic, fabric, glass, and wood. Nowadays, these materials have largely been replaced by large machines that incorporate various types of plastics in their composition. Examples include plastic bags and/or rigid baskets used in olive harvesting, conveyor belt bases, hoses for transporting the product along the production line, tanks, sealing rings (O-rings), unions, and sealing plugs, among others. Even in storage, stainless steel tanks are commonly used, but plastic tanks are also employed. Likewise, in the final packaging stage, plastic bottles are often used instead of glass.

Thus, the contamination of olive oil with plasticizers added to plastics can occur at various stages of the production chain, with packaging contact being, in most cases, only the final stage. This means that packaging may not necessarily be the primary source of plasticizers found in olive oil.

The contamination occurs because plasticizers do not chemically bind to the polymer matrix, which allows them to migrate over time due to factors such as exposure, increased temperature, and mechanical stress, among others [47].

To determine the sources of plasticizer contamination, samples from different stages across three olive oil production lines in Portugal, as well as samples of packaged and stored olive oil (Figure 1), were analyzed using the previously described analytical method.

The quantities of all plasticizers analyzed and detected in the samples are reported in Table S1 of the Supplementary Material. Table 2 summarizes the data specifically for the concentrations of DIBP, DBP, BBP, DEHP, and the sum of DINP and DIDP, selected based on the European Commission Regulation (2023/1442). Additionally, the table includes the total sum of all plasticizers involved in the study, as defined under Restriction Group No. 32 of the same regulation, which establishes a specific migration limit of 60 mg/kg for the combined levels of DBP, BBP, DEHP, DIBP, and other plasticizing substances. For plasticizers found under the quantification limit (<LOQ), this value was added to the sum to obtain a “worst-case scenario”.

It is important to emphasize that this regulation pertains only to migration limits for materials in contact with food and does not define the allowable limits of these substances in the food itself.

All regulated phthalates were detected at least once along the production lines. BBP was only found in two production lines and only in a single sample from northern Portugal above LOQ. DBP, on the other hand, was primarily detected in the Central production line. But it was in the North line that the concentration of DBP in olive oil packaged in PET for 18 months slightly exceeded the specific migration limit established by European regulations. DEHP, however, was almost always below the LOD across all production lines. DIBP was detected in all lines but always at concentrations below 0.093 mg/kg. DINP and DIDP stood out as the main contributors to the increase in plasticizers along the production lines, with concentrations exceeding the specific migration limits established by Regulation (EU) 2023/1442 for the sum of these two phthalates (1.8 mg/kg). DINP, known for replacing DEHP in many industrial applications, was the most abundant compound overall (see Table S1 in the Supplementary Material) [48]. 

Similar results were reported by Nanni et al., who investigated 172 samples of vegetable oils marketed in Italy, including olive oil. In their study, DINP was also identified as the plasticizer present at the highest levels in olive oils, with an average concentration of 1.7 mg/kg in extra virgin olive oils and 2.9 mg/kg in regular olive oils [49]. Likewise, Pereira et al. detected DINP in European olive oil samples, reporting it as one of the phthalates with the highest concentrations. Their study found an average DINP concentration of 1.5 mg/kg across samples, with a maximum value of 6.29 mg/kg [50]. Similarly, Arena et al. observed comparable results, with DINP concentrations ranging from 2.4 to 7.60 mg/kg in extra virgin olive oils [51].

Regarding the sum of the 32 plasticizers analyzed, no sample exceeded the specific migration limits established by Regulation (EU) for Restriction Group No. 32, which stipulates that the sum of DBP, BBP, DEHP, DIBP, and other plasticizing substances such as adipates, sebacates, and terephthalates, among others, must not exceed 60 mg/kg.

In addition to the regulated phthalates, other phthalates and plasticizers contributed significantly to the total values observed, particularly in packaged olive oils. Among these, the most notable were DMEP, with concentrations ranging from 0.969 to 4.342 mg/kg; DPP, between 0.005 and 9.818 mg/kg; DEHT, with values between 0.153 and 8.538 mg/kg (see Table S1 in the Supplementary Material).

Although these compounds are not specifically regulated in the European Union’s table of SMLs for phthalates, they represent a significant contribution to the total load of plasticizers detected. DMEP is widely used as a solvent and plasticizer, particularly in paints and resins. Despite its relatively low toxicity compared to other phthalates, its presence in food warrants attention due to potential migration from contact materials [52]. DPP, though less studied, is used in industrial applications and exhibits low volatility, which may favor its accumulation [53]. Meanwhile, DEHT is often employed as an alternative to more toxic phthalates and is considered a low-risk plasticizer in food-related applications [54]. However, recent studies suggest that even plasticizers deemed safe may pose potential long-term risks due to cumulative exposure [55].

Additionally, it is crucial to note that the total plasticizer values reported in this study reflect only the 32 compounds analyzed, while hundreds of plasticizers are currently available in the market, whose presence and impact on food remain underexplored.

These findings highlight the importance of monitoring not only the regulated plasticizers but also widely used substitutes, ensuring a comprehensive assessment of the human health risks associated with their presence in food.

**Figure 2:**
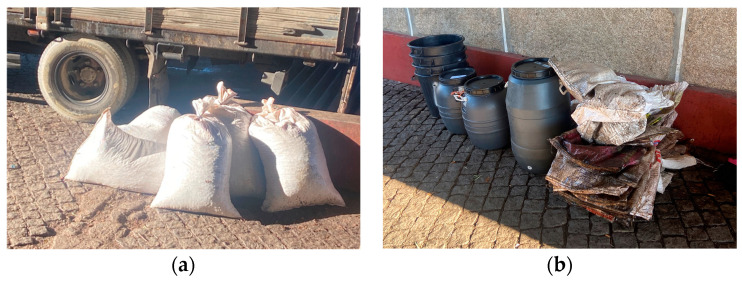
Plastic bags used for olive transport in the northern production line. Photo (**a**) shows bags filled with olives and photo (**b**) shows empty bags and containers used to transport the olives.

The three olive oil production lines from northern, central, and southern Portugal exhibited distinct patterns regarding the presence of plasticizers, making it difficult to identify the main sources of contamination. In the Northern production line, olives were analyzed before cleaning, and it was observed that those transported in reusable plastic bags (Figure 2) were already contaminated upon arrival, albeit at low levels. After the olives were cleaned with water, a reduction in these contaminants was observed, suggesting that washing partially removes plasticizer particles originating from the bags.

In the Central line, the levels of plasticizer contamination in the olives were higher from the start than in the Northern and Southern lines. Interestingly, in the Southern line, no plasticizers were quantified in the unprocessed olives. Nevertheless, contamination accumulated throughout the production process, similar to what was observed in the Central line. On the other hand, the Northern line appeared to contribute the least to olive oil contamination up to the storage stage.

**Figure 3:**
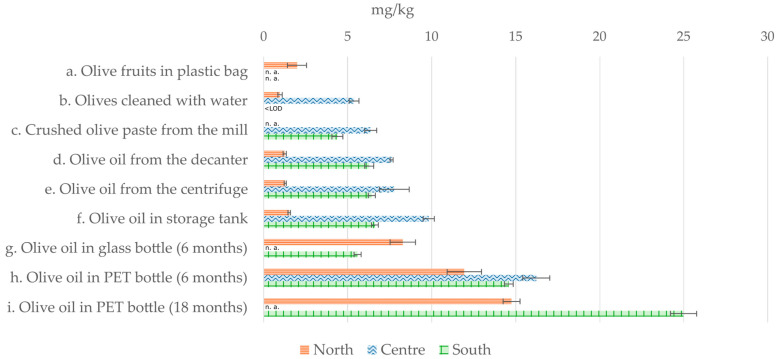
Graphical representation of the sum of the 32 plasticizers analyzed across the three production lines. n. a.—not analyzed.

Overall, a progressive increase in plasticizer contamination was observed along the production lines (Figure 3).

**Figure 4:**
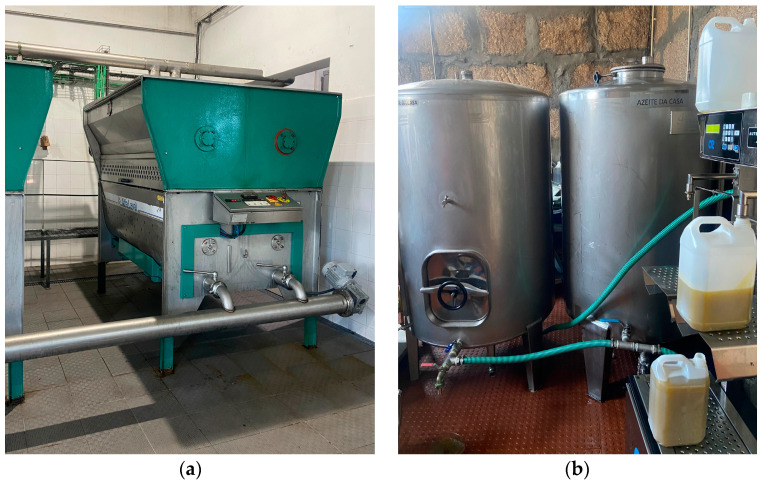
Production line equipped with stainless steel tubes versus plastic hoses. Photo (**a**) shows steel tubing, whereas (**b**) shows plastic hoses.

This increase may be associated with the equipment, tools, and containers used during production. However, the most significant increment in contamination was observed during storage and packaging. During sample collection in all production lines, it was noted that the crushed olive paste and olive oil were transported mainly through stainless steel tubes between the mill mixer, the decanter, and the centrifuge, as well as between the centrifuge and the storage tanks (which were made of either stainless steel or rigid plastic). However, during the bottling process (in glass or plastic bottles), the use of plastic hoses to transport the olive oil was identified (Figure 4).

This detail suggests that the primary contamination source is not directly related to the material of bottles but rather to the hoses used during transportation to the packaging stage.

Another relevant observation was made when comparing olive oil packaged in plastic bottles and stored for 6 months versus 18 months in the Northern and Southern lines. A considerable increase in plasticizer concentrations was observed over time, likely due to prolonged contact between the olive oil and the packaging, resulting in greater migration of plasticizer compounds. The Southern line showed a more significant increase, suggesting differences in the PET composition used across the production lines. These results emphasize the importance of carefully selecting the appropriate packaging material to minimize contamination during storage, as well as not prioritizing plastic bottles for long-term storage.

### 2.3. Off-the-Shelf Olive Oil

In the previously investigated production lines, only two of them used glass bottles as packaging material in addition to PET. Although an increase in plasticizer concentrations was observed in both lines during storage, their behaviors differed. In the production line in the North, the difference in plasticizer concentrations between olive oil packaged in glass and olive oil packaged in PET was smaller compared to the production line in the South. This observation highlighted the need for a more detailed analysis of the impact of different packaging materials, considering their potential influence on plasticizer contamination levels. 

In order to test these findings, samples of olive oil from the same brand, packaged in glass bottles and PET containers, were purchased from a local supermarket. Sample identification and the results of the analyzed plasticizers are presented in Table 3.

A slight increase in plasticizer levels was observed in the samples packaged in PET compared to those in glass. Among the three analyzed samples, plasticizer levels varied significantly, with olive oil two exhibiting the lowest plasticizer concentrations compared to the other two olive oils. Consistent with the results observed in the production lines, the sum of DINP and DIDP represented the main contribution to plasticizer contamination, with concentrations exceeding the specific migration limits established by Regulation (EU) 2023/1442.

Additionally, three other oils were analyzed, two packaged in glass and one in a metal can, which showed plasticizer levels within different ranges from the previously analyzed samples. The oil packaged in a metal can exhibited the lowest plasticizer concentrations among all the samples studied, but it was also the only one not produced or packaged in Portugal.

Despite the differences observed between oils packaged in glass and PET, these were minor and consistent with findings from other published studies [47,49,50,56]. For instance, Bi et al. studied edible oils, including olive oil, in the United States and found no significant differences in plasticizer concentrations among glass, plastic, and metal packaging, leading the authors to conclude that packaging is not the primary source of contamination [27]. Similarly, a European study also concluded that the presence of phthalates in olive oil is not necessarily associated with plastic packaging after comparing various packaging materials [50].

These results reinforce the hypothesis that the main source of plasticizer contamination may not be exclusively related to the type of packaging but rather to the widespread use of plastic materials throughout the production process. Additionally, the impact of environmental factors on food contamination with plasticizers should also be considered, an aspect that warrants further investigation.

## 3. Experimental Section

### 3.1. Chemicals

Thirty-two plasticizers were analyzed: dimethyl phthalate (DMP), diethyl phthalate (DEP), diallyl phthalate (DAP), dipropyl phthalate (DPrP), diisobutyl phthalate (DIBP), dibutyl phthalate (DBP), bis(2-methoxyethyl) phthalate (DMEP), diisopentyl phthalate (DIPP), bisphenol A (BPA), benzyl butyl phthalate (BBP), dihexyl phthalate (DHXP), dicyclohexyl phthalate (DCHP), diphenyl phthalate (DPhP), bis(2-ethylhexyl) phthalate (DEHP), di-n-heptyl phthalate (DHP), dioctyl phthalate (DOP), di(2-ethylhexyl) terephthalate (DEHT), diisononyl phthalate (DINP), and diisodecyl phthalate (DIDP), which were acquired from Sigma-Aldrich (Steinheim, Germany). Additionally, dimethyl terephthalate (DMTP), dibutyl maleate (DBM), diisopropyl phthalate (DiPrP), diethyl sebacate (DES), bis(4-methyl-2-pentyl) phthalate (BMPP), bis(2-ethoxyethyl) phthalate (DEEP), dipentyl phthalate (DPP), acetyltributyl citrate (ATBC), bis(2-ethylhexyl) adipate (DEHA), bis(2-n-butoxyethyl) phthalate (DBEP), di(2-ethylhexyl) sebacate (DEHS), dinonyl phthalate (DNP), tris(2-ethylhexyl) trimellitate (TOMT), and the internal standard Benzyl Butyl Phthalate-d4 were purchased from Dr. Ehrenstorfer GmbH (Augsburg, Germany). Acetone, hexane, and methanol of GC-MS grade were obtained from Carlo Erba (Emmendingen, Germany). 

Stock solutions for each plasticizer were prepared in acetone at 500 µg/mL and stored at 4 °C for at most one month.

As certified olive oil without plasticizers was not commercially available, an organic virgin sunflower oil with no detectable plasticizers was used for method validation. This oil was previously tested for plasticizer presence and content to ensure it could be considered suitable for matrix effect simulation. When residual contamination was detected in blank injections, it was subtracted from the sample results.

All solvents used for sample preparation were analyzed daily for the presence of plasticizers. Only glassware lab material was used, which was carefully washed, rinsed, and stored at 100 °C before use.

Additionally, the chromatographic system was checked daily for plasticizers by performing three blank injections at the start, during, and at the end of analyses.

### 3.2. Sampling

Samples of olives, olive paste, and olive oil at different steps of the production process were collected from three olive oil production lines located in different regions of Portugal (North, Center, and South). These were stored in glass containers with non-plastic lids, namely bamboo and glass, and frozen until analysis. Olive oil at the end of each production line was collected in both glass and PET containers and stored at room temperature to simulate normal shelf conditions. These containers were supplied by each production line. 

Olive samples were processed into olive oil in the laboratory, firstly using an IKA-Werke A 10 stainless-steel grinder (Staufen, Germany). The paste was then transferred to glass culture tubes, heated to around 50 °C, and centrifuged at 3000 RPMs until sufficient oil was separated. This was removed and immediately weighed for analysis.

Off-the-shelf extra virgin olive oils, in both glass and plastic bottles, were purchased from a local supermarket.

### 3.3. Sample Analysis and Method Validation

The analytical method was adapted from a protocol TFDAA0008.02 established by the Taiwan Food and Drug Administration (TFDA) for testing phthalate plasticizers in foods. The method was optimized based on the extraction solvents used, the amount of solvent used, and the speed and timing of the vortex mix and centrifugation. Additionally, a freezing step was added to the procedure.

A 500 mg oil sample was weighed into a 15 mL glass test tube, and then 100 μL of internal standard (IS) (2.5 mg/L) was added and vortexed for 30 s. Then, 0.3 mL of hexane and 2 mL of hexane/methanol (1:4, *v*/*v*) were added, vortexed again for 1 min, and centrifuged at 2000 RPMs for 2 min. A 1.2 mL aliquot of the supernatant was carefully removed, after which another 2 mL of hexane/methanol (1:4, *v*/*v*) was added to the tube, vortexed 1 min, and centrifuged as above. A total of 2 mL of the supernatant was removed and added to the previous one for a total volume of 3.2 mL. This extract was then frozen at −24 °C overnight.

With the sample still frozen, an aliquot of the liquid phase was quickly transferred to a glass vial, allowed to reach room temperature, and injected into the chromatographic system. For the blanks, the entire procedure was performed with unspiked sunflower oil. Three replicates were performed for each sample.

Standard calibration solutions were prepared by spiking the sunflower oil at 0.001–16 mg/kg for all plasticizers. When linearity was not observed throughout the calibration range, two different regression curves were constructed.

Limits of detection (LOD) and quantification (LOQ) were determined considering that the lowest calibration concentration for each compound with a signal-to-noise ratio greater than 3 was the experimental LOD, and greater than 10 was the LOQ.

### 3.4. Chromatographic Conditions for GC-MS/MS

Analyses were performed on a Bruker (Bremen, Germany) GC 456 coupled with a Bruker Scion TQ (Triple Quadrupole) system equipped with a CTC (Zwingen, Switzerland) CombiPAL autosampler. Data acquisition was managed using Bruker MSWS 8.2 software, and analysis was conducted with Bruker MS Data Review 8.0. Chromatographic separation was achieved with a ZB-5MS Plus capillary column (20 m × 0.18 mm ID, 0.18 µm film thickness) supplied by Phenomenex (Torrance, CA, USA). The oven temperature program started at 50 °C, held for 1 min, increased at 20 °C/min to 140 °C, then 4 °C/min to 240 °C, followed by 10 °C/min to 280 °C, and finally 20 °C/min to 310 °C, where it was held for 9 min.

High-purity helium (99.9999%) was used as the carrier gas at a constant flow rate of 0.7 mL/min, with an injection volume of 1 µL. The mass spectrometer was operated in multiple reaction monitoring (MRM) mode, using argon as the collision gas at 2.4 mTorr. The transfer line was maintained at 300 °C, and the ion source at 270 °C. A solvent delay of 7 min was applied.

The MRM transitions, associated with selected precursor and product ion pairs for each analyte, are listed in Table 4. Quadrupoles operated at unit resolution, and ion ratios between the quantifier and qualifier ions were required to be within ± 30% of the average standard injections for positive identification [57]. Determination coefficients (R^2^) obtained for all compounds were between 0.958 and 0.998. 

## 4. Conclusions

This study investigated the sources of plasticizer contamination in olive oil. The optimized analytical method used for quantification demonstrated adequate performance in terms of detection limits and excellent repeatability while requiring relatively small solvent volumes and ensuring effective sample clean-up. Analyses conducted throughout the production line revealed a progressive increase in plasticizer concentrations, having identified olive harvesting and industrial processes as predominant contamination sources, along with storage, particularly in PET packaging, over long periods.

Initial contamination in olives may have been influenced by factors such as the use of plastic nets and bags, as well as by the metabolism of the olive tree, which facilitates the absorption of compounds from soil, water, and air.

Among the compounds analyzed, DINP was the most frequent, with an average concentration of 3.387 mg/kg and a maximum value of 9.393 mg/kg in oils stored in both glass and PET. These results indicate that some stored oils exceeded the specific migration limits established by European regulations (1.8 mg/kg). The significant presence of DINP, as opposed to plasticizers like DEHP, reflects the gradual replacement of the latter in industrial applications and highlights the growing prevalence of DINP in construction materials, industrial machinery, and ecosystems.

Although the total concentration of plasticizers analyzed did not exceed the limit set by European regulations (60 mg/kg), it should be noted that this study covered only 32 compounds, whereas many other plasticizers are currently in use.

Given the importance of olive oil as a widely consumed food product, it is essential to precisely identify contamination sources and implement effective mitigation strategies. Replacing plastic materials with safer alternatives, such as stainless steel or adopting phthalate-free plastics, are fundamental measures. However, it is equally crucial to monitor and evaluate these materials, as even those labeled as phthalate-free may release contaminants over time due to mechanical stress and temperature. Additionally, new phthalate replacement plasticizers must be monitored and toxicologically tested.

Accurate diagnostics and the implementation of mitigation strategies will significantly reduce plasticizer contamination, ensuring greater consumer safety and preserving the quality of this essential food product.

## Figures and Tables

**Table 1:** Phthalates permitted in food contact materials by regulation (EU) 2023/1442, their SML and intended uses.

Substance	Regulation (EU) 2023/1442 Amending Annex I to Regulation (EU) 10/2011	Only to Be Used as:
Dibutyl Phthalate (DBP)	SML: 0.12 mg/kg Total SML group restriction no. 32: 60 mg/kg Total SML group restriction no. 36: 0.6 mg/kg	(a) Plasticizer in repeated use materials and articles contacting non-fatty foods; (b) Technical support agent in polyolefins in concentrations up to 0.05% (*w*/*w*) in the final product.
Benzyl Butyl Phthalate (BBP)	SML: 6.0 mg/kg Total SML group restriction no. 32: 60 mg/kg Total SML group restriction no. 36: 0.6 mg/kg	(a) Plasticizer in repeated use materials and articles; (b) Plasticizer in single-use materials and articles contacting non-fatty foods except for infant formula and follow-on formula; (c) Technical support agent in concentrations up to 0.1% (*w*/*w*) in the final product.
Di(2-Ethylhexyl) Phthalate (DEHP)	SML: 0.6 mg/kg Total SML group restriction no. 32: 60 mg/kg Total SML group restriction no. 36: 0.6 mg/kg	(a) Plasticizer in repeated use materials and articles contacting non-fatty foods; (b) Technical support agent in concentrations up to 0.1% (*w*/*w*) in the final product.
Di-isononyl Phthalate and Di-isodecyl Phthalate (DINP and DIDP)	Total SML group restriction no. 26: 1.8 mg/kg (sum of DINP and DIDP) Total SML group restriction no. 32: 60 mg/kg Not to be used in combination with FCM substances DBP, BBP, DEHP, and DIBP.	(a) Plasticizer in repeated use materials and articles; (b) Plasticizer in single-use materials and articles contacting non-fatty foods except for infant formula and follow-on formula; (c) technical support agent in concentrations up to 0.1% (*w*/*w*) in the final product.

Group restriction no. 26 corresponds to the sum of DINP e DIDP; Group restriction no. 36 corresponds to the sum of DBP, DIBP, BBP, and DEHP expressed as DEHP equivalents using the following equation: DBP × 5 + DIBP × 4 + BBP × 0.1 + DEHP × 1; Group restriction no. 32 corresponds to the sum of DBP BBP DEHP DIBP and some plasticizing substances like adipates, sebacates, and terephthalates, among others. DIBP is not listed as an authorized substance; however, it may occur alongside other phthalates as a result of its use as a polymerization aid, and therefore, it is included in group restrictions.

**Table 2:** Concentration (mg/kg) of phthalates regulated by Regulation (EU) 2023/1442 and the sum of the 32 plasticizers studied in samples collected from the three production lines, expressed as average ± standard deviation. For the sums of analytes, the standard deviation was calculated by the square root of the sum of variances. Bold and underlined values indicate those exceeding the specific migration limits defined. n. a.—not analyzed.

	Samples of Production Line	DIBP	DBP	BBP	DEHP	Sum of DINP and DIDP	Sum of 32 Plasticizers
**NORTH**	a. Olive fruits in plastic bag	0.011 ± 0.004	<LOD	<LOD	<LOD	<LOD	1.987 ± 0.571
	b. Olives cleaned with water	0.008 ± 0.001	<LOD	<LOD	<LOD	<LOD	0.971 ± 0.133
	c. Crushed olive paste from the mill mixer	n. a.					
	d. Olive oil from the decanter	<LOD	<LOQ	<LOQ	<LOD	<LOD	1.253 ± 0.100
	e. Olive oil from the centrifuge	0.007 ± 0.002	<LOQ	<LOQ	<LOD	<LOD	1.277 ± 0.066
	f. Olive oil in storage tank	<LOD	<LOD	<LOQ	<LOD	0.103 ± 0.061	1.522 ± 0.071
	g. Olive oil in glass bottle (6 months)	<LOD	<LOD	<LOQ	<LOD	1.278 ± 0.161	8.277 ± 0.752
	h. Olive oil in PET bottle (6 months)	0.019 ± 0.008	<LOQ	<LOQ	<LOD	**3.527 ± 0.214**	11.946 ± 1.028
	i. Olive oil in PET bottle (18 months)	0.028 ± 0.009	**0.127 ± 0.007**	0.006 ± 0.001	0.454 ± 0.013	**6.000 ± 0.203**	14.751 ± 0.506
**CENTRE**	a. Olive fruits in plastic bag	n. a.					
	b. Olives cleaned with water	0.019 ± 0.010	0.073 ± 0.004	<LOD	0.076 ± 0.007	0.146 ± 0.056	5.377 ± 0.289
	c. Crushed olive paste from the mill mixer	0.052 ± 0.010	0.085 ± 0.004	<LOD	<LOD	0.431 ± 0.145	6.372 ± 0.359
	d. Olive oil from the decanter	0.061 ± 0.007	0.083 ± 0.012	<LOD	<LOD	0.318 ± 0.065	7.615 ± 0.086
	e. Olive oil from the centrifuge	0.079 ± 0.013	0.082 ± 0.007	<LOD	<LOD	0.625 ± 0.309	7.780 ± 0.888
	f. Olive oil in storage tank	0.093 ± 0.008	0.092 ± 0.002	<LOQ	<LOD	1.211 ± 0.172	9.821 ± 0.331
	g. Olive oil in glass bottle (6 months)	n. a.					
	h. Olive oil in PET bottle (6 months)	0.072 ± 0.007	0.096 ± 0.009	<LOQ	<LOD	**3.528 ± 0.323**	16.224 ± 0.807
	i. Olive oil in PET bottle (18 months)	n. a.					
**SOUTH**	a. Olive fruits in plastic bag	n. a.					
	b. Olives cleaned with water	<LOD	<LOD	<LOD	<LOD	<LOD	<LOD
	c. Crushed olive paste from the mill mixer	0.013 ± 0.006	<LOQ	<LOD	<LOD	<LOQ	4.385 ± 0.330
	d. Olive oil from the decanter	0.010 ± 0.008	<LOQ	<LOD	<LOD	0.103 ± 0.023	6.285 ± 0.271
	e. Olive oil from the centrifuge	0.010 ± 0.002	<LOD	<LOD	<LOD	0.156 ± 0.015	6.429 ± 0.212
	f. Olive oil in storage tank	<LOQ	<LOD	<LOD	<LOD	0.264 ± 0.064	6.621 ± 0.200
	g. Olive oil in glass bottle (6 months)	<LOQ	<LOQ	<LOD	0.037 ± 0.003	**4.396 ± 0.156**	5.590 ± 0.215
	h. Olive oil in PET bottle (6 months)	<LOQ	<LOQ	<LOD	0.078 ± 0.009	**5.112 ± 0.228**	14.595 ± 0.262
	i. Olive oil in PET bottle (18 months)	<LOD	0.038 ± 0.002	<LOD	0.095 ± 0.001	**9.393 ± 0.580**	24.991 ± 0.786

**Table 3:** Concentration (mg/kg) of phthalates regulated by Regulation (EU) 2023/1442 and the sum of the 32 plasticizers studied in olive oils purchased from local supermarket. Expressed as average ± standard deviation. For the sums of analytes, the standard deviation was calculated by the square root of the sum of variances. Bold and underlined values indicate those exceeding the specific migration limits defined.

		DIBP	DBP	BBP	DEHP	Sum of DINP and DIDP	Sum of 32 Plasticizers
Olive Oil 1	GLASS	0.014 ± 0.009	<LOD	<LOD	<LOD	1.598 ± 0.145	2.688 ± 0.146
	PET	<LOQ	<LOD	<LOD	<LOD	**1.807 ± 0.199**	4.109 ± 0.204
							
Olive Oil 2	GLASS	<LOQ	0.020 ± 0.005	<LOD	<LOD	0.395 ± 0.076	1.168 ± 0.116
	PET	0.018 ± 0.005	<LOQ	<LOD	<LOD	0.716 ± 0.112	1.516 ± 0.117
							
Olive Oil 3	GLASS	<LOD	<LOD	<LOD	<LOD	**3.310 ± 0.053**	5.763 ± 0.122
	PET	<LOQ	<LOD	<LOQ	<LOD	**3.245 ± 0.050**	5.847 ± 0.131
							
Olive Oil 4	GLASS	<LOD	<LOD	<LOQ	**0.656 ± 0.006**	1.244 ± 0.086	3.299 ± 0.093
Olive Oil 5	GLASS	0.02 ± 0.002	<LOD	<LOD	<LOD	**5.976 ± 0.389**	7.145 ± 0.391
Olive Oil 6	CAN	<LOD	<LOD	<LOD	0.082 ± 0.013	<LOD	0.882 ± 0.056

**Table 4:** MRM parameters for the analysis of the 32 plasticizers, as well as detection and quantification limits (LOD and LOQ, respectively).

Plasticizers	CAS	Quantifier Transition (eV)	Qualifier Transition (eV)	LOD (mg/kg)	LOQ (mg/kg)
DMP	131-11-3	163 > 77 (14)	163 > 92 (28)	0.002	0.007
DMTP	120-61-6	163 > 75 (30)	163 > 103 (18)	0.005	0.018
DBM	105-76-0	117 > 99 (10)	117 > 71 (16)	0.001	0.004
DEP	84-66-2	149 > 65 (22)	149 > 121 (14)	0.005	0.018
DiPrP	605-45-8	149 > 65 (24)	149 > 121 (16)	0.001	0.004
DAP	131-17-9	149 > 65 (22)	149 > 121 (14)	0.013	0.043
DPrp	131-16-8	149 > 65 (24)	149 > 121 (14)	0.005	0.018
DES	110-40-7	171 > 55 (23)	171 > 97 (12)	0.031	0.103
DIBP	84-69-5	149 > 65 (24)	149 > 121 (16)	0.002	0.007
DBP	84-74-2	149 > 65 (24)	149 > 121 (16)	0.005	0.018
DMEP	117-82-8	149 > 65 (24)	149 > 121 (16)	0.103	0.343
BMPP	84-63-9	149 > 65 (24)	251 > 149 (15)	0.005	0.018
DIPP	605-50-5	149 > 65 (24)	237 > 149 (12)	0.005	0.018
DEEP	605-54-9	149 > 65 (22)	149 > 121 (14)	0.005	0.018
DPP	131-18-0	149 > 65 (24)	149 > 121 (16)	0.001	0.004
BPA	80-05-7	231 > 91 (28)	119 > 91 (14)	0.031	0.103
ATBC	77-90-7	129 > 69 (18)	185 > 69 (24)	0.001	0.004
BBP	85-68-7	149 > 65 (24)	238 > 149 (18)	0.001	0.004
DHXP	84-75-3	149 > 65 (24)	251 > 149 (14)	0.001	0.004
DEHA	103-23-1	129 > 55 (16)	129 > 111 (17)	0.005	0.018
DBEP	117-83-9	149 > 65 (22)	149 > 121 (14)	0.031	0.103
DCHP	84-61-7	149 > 65 (24)	167 > 149 (10)	0.005	0.018
DPhP	84-62-8	225 > 77 (22)	225 > 51 (50)	0.001	0.004
DEHP	117-81-7	149 > 65 (20)	279 > 149 (18)	0.005	0.018
DHP	3648-21-3	149 > 65 (24)	265 > 149 (15)	0.009	0.030
DOP	117-84-0	149 > 65 (24)	149 > 121 (16)	0.001	0.004
DEHT	6422-86-2	149 > 65 (19)	167 > 79 (14)	0.005	0.018
DEHS	122-62-3	185 > 69 (16)	203 > 121 (14)	0.005	0.018
DNP	84-76-4	149 > 65 (24)	149 > 121 (16)	0.005	0.018
DINP	28553-12-0	293 > 149 (5)	293 > 71 (5)	0.031	0.103
DIDP	26761-40-0	307 > 149 (5)	307 > 71 (5)	0.103	0.343
TOMT	3319-31-1	305 > 193 (20)	193 > 81 (26)	0.005	0.018

## Data Availability

Data are contained within the article.

## Supplementary Materials

The following supporting information can be downloaded at https://www.mdpi.com/article/10.3390/molecules29246013/s1, Table S1: Concentration (mg/kg) of the 32 plasticizers studied in all collected samples, expressed as average ± standard deviation. Bold and underlined values indicate those exceeding the specific migration limits defined.
